# Bridging the Gap between Gut Microbiota and Alzheimer’s Disease: A Metaproteomic Approach for Biomarker Discovery in Transgenic Mice

**DOI:** 10.3390/ijms241612819

**Published:** 2023-08-15

**Authors:** Esra Ayan, Hasan DeMirci, Muhittin Abdulkadir Serdar, Francesca Palermo, Ahmet Tarık Baykal

**Affiliations:** 1 Department of Biochemistry and Molecular Biology, Faculty of Medicine, Acibadem Mehmet Ali Aydinlar University, Istanbul 34450, Turkey; esraayan20@ku.edu.tr (E.A.); muhittin.serdar@acibadem.edu.tr (M.A.S.); 2 Department of Molecular Biology and Genetics, Faculty of Science, Koç University, Istanbul 34450, Turkey; hdemirci@ku.edu.tr; 3 Koç University Isbank Center for Infectious Diseases (KUISCID), Koç University, Istanbul 34450, Turkey; 4 Stanford PULSE Institute, SLAC National Laboratory, Menlo Park, CA 94305, USA; 5 Institute of Nanotechnology—CNR, Rome Unit, 00185 Rome, Italy; francesca.palermo00@gmail.com; 6 Acıbadem Labmed Clinical Laboratories, R&D Center, İstanbul 34450, Turkey; 7 Department of Medical Biochemistry, Faculty of Medicine, Acibadem Mehmet Ali Aydınlar University, Istanbul 34450, Turkey

**Keywords:** neurodegeneration, Alzheimer’s Disease, proteomics, metaproteomics, gut microbiota, 5xFAD mice

## Abstract

Alzheimer’s Disease (AD) is a progressively debilitating form of dementia that affects millions of individuals worldwide. Although a vast amount of research has investigated the complex interplay between gut microbiota and neurodegeneration, the metaproteomic effects of microbiota on AD pathogenesis remain largely uncharted territory. This study aims to reveal the role of gut microbiota in AD pathogenesis, particularly regarding changes in the proteome and molecular pathways that are intricately linked to disease progression. We operated state-of-the-art Nano-Liquid Chromatography Mass Spectrometry (nLC-MS/MS) to compare the metaproteomic shifts of 3-month-old transgenic (3M-ALZ) and control (3M-ALM, Alzheimer’s Littermate) mice, depicting the early onset of AD with those of 12-month-old ALZ and ALM mice displaying the late stage of AD. Combined with computational analysis, the outcomes of the gut–brain axis-focused inquiry furnish priceless knowledge regarding the intersection of gut microbiota and AD. Accordingly, our data indicate that the microbiota, proteome, and molecular changes in the intestine arise long before the manifestation of disease symptoms. Moreover, disparities exist between the normal-aged flora and the gut microbiota of late-stage AD mice, underscoring that the identified vital phyla, proteins, and pathways hold immense potential as markers for the early and late stages of AD. Our research endeavors to offer a comprehensive inquiry into the intricate interplay between gut microbiota and Alzheimer’s Disease utilizing metaproteomic approaches, which have not been widely adopted in this domain. This highlights the exigency for further scientific exploration to elucidate the underlying mechanisms that govern this complex and multifaceted linkage.

## 1. Introduction

Over the past decade, studies have demonstrated the co-presence of various microorganisms, including bacteria, fungi, parasites, and viruses, within the human intestine. In fact, the number of microorganisms in our body is estimated to be 10–100 times greater than the number of eukaryotic cells [1]. These microorganisms, which live in symbiosis with the human host, are collectively referred to as microbiota. Note that the term microbiota is distinct from the term microbiome, which encompasses all the genes of the microbiota [2,3]. The composition of the gut microbiota is typically dominated by several key phyla, including *Firmicutes, Bacteroidetes, Proteobacteria, Actinomycetes, Verrucomicrobia,* and *Fusobacteria* [4,5]. While the specific makeup of an individual’s microbiota may vary, evidence suggests that all individuals possess a core microbiota [6].

Recently, the term “Gut–Brain Axis” has gained prominence in scientific literature to describe the bidirectional communication between the Central Nervous System (CNS) and Enteric Nervous System (ENS). Evidence suggests that this axis plays a critical role in various stages of life, from early development to old age, and in different body regions, from the intestinal lumen to the CNS [7]. However, understanding the mechanisms involved in this bidirectional communication is essential to advance our understanding of this axis [8]. The Gut–Brain Axis involves various physiological pathways, including the nervous, immune, endocrine, and metabolic signals [7,8]. Within the nervous system pathway, the ENS, primarily located within the gastrointestinal tract, modulates signals through both sympathetic and parasympathetic pathways, mainly through the vagus nerve and prevertebral ganglia. Studies in germ-free mice have provided compelling evidence that gut microbiota plays a critical role in the neural development of young and adult brains [8,9]. Thus, it is becoming increasingly evident that gut microbiota alterations can trigger the disease process’s onset [10,11]. Therefore, a more comprehensive understanding of the Gut–Brain Axis and the mechanisms underlying its interactions could pave the way for developing novel therapeutic interventions for various neurological and psychiatric disorders.

Neurodegenerative disorders have been recognized to manifest with aging and are associated with heightened susceptibility, which decreased intestinal microbial diversity and altered microbiota composition associated with aging [12]. Consequent to immune system dysregulation, these conditions lead to chronic intestinal inflammation and microbial dysbiosis [7]. Furthermore, microbial products traverse the blood–brain barrier through circulation and activate pro-inflammatory cytokines via microglial activity, leading to neuroinflammation in the brain [7,13]. Studies in germ-free mice have revealed that bacterial short-chain fatty acids (SCFAs) considerably impact microglial maturation and the tryptophan pathway, which affects astrocyte activation; this is a microbial influence on neurogenesis [14]. From this vantage point, it becomes apparent that the gut microbiota significantly impacts the immune and lymphatic systems via the bidirectional Gut–Brain Axis and neurogenesis [7,9,15]. Additionally, the gut microbiota is accountable for the biosynthesis of crucial neuromodulators and neurotransmitters, including serotonin and dopamine [16,17]. This is of utmost importance as these signals have a critical role in enterochromaffin cells, a subset of enterocytes that function as ENS neurotransmitters responsible for the integrity of the intestinal epithelium [16,17]. Hence, it can be posited that Gut–Brain Axis dysbiosis exerts a noteworthy influence on the pathogenesis of neurodegeneration, including Alzheimer’s Disease (AD) [7,18]. Additionally, emerging evidence from Irritable Bowel Syndrome and other recent investigations have elucidated the potential interplay between the host’s innate immune system and gut microbiota. Consequently, intestinal microbiota dysbiosis is a notable risk factor for AD or non-AD dementia. Furthermore, dysbiosis of the intestinal microbiota can undermine the host immune response, leading to the progression of various inflammatory disorders such as autism, schizophrenia, and multiple sclerosis [7,9,19,20].

AD is a prevalent neurodegenerative disorder that exerts deleterious effects on cognitive abilities [21]. AD is typified by the deposition of extracellular amyloid–beta (Aβ) aggregates known as senile plaques and intracellular neurofibrillary tangles of hyperphosphorylated microtubule-associated tau (MAPT) protein [22]. The 5XFAD is a transgenic mouse model notable for its early-onset and robust Aβ overexpression [23]. These mice exhibit intracellular Aβ accumulations in various subcortical and cortical regions, particularly in the hippocampus, resulting in AD-like cognitive impairments within 2–4 months [24]. After approximately 4 months, marked gliosis and amyloid plaque deposits are observed [25]. The selection of this model was informed by the fact that it presents a vast quantity of toxic Aβ plaques, which disrupt synaptic junction networks, resulting in a degenerative phenotype characterized by neuronal atrophy and death without the manifestation of tau pathology [26,27]. Additionally, this model organism has been employed in numerous gut–brain axis studies, and its relevance to this area of research is well-documented in the scientific literature [28,29,30]. Despite significant efforts to develop effective treatments for AD, recent years have witnessed consistent failures of tau-targeted or β-amyloid (Aβ)-centered medical strategies in late-onset clinical trials [31,32]. Collective studies from both in vivo and in vitro settings, including human and animal studies, have demonstrated a strong association between intestinal microbiota dysbiosis and inflammatory responses, including microglial and astrocyte activation, throughout the development of AD [33,34,35]. Notably, numerous studies have shown plaque-related inflammatory activity in mice treated with antibiotics, the presence of intestinal microbial lipopolysaccharide (LPS) in post-mortem brain analyses of AD patients, and the activation of peripheral inflammation upon LPS injection [36,37,38].

The present knowledge regarding the association between the gut–brain axis and AD pathogenesis needs to be completed [7,39]. The precise inflammatory mechanisms interconnected during AD development remain elusive [39,40]. Additionally, the significance of microbiota-derived metabolites in AD-linked pathways involving the gut–brain axis and neuroinflammation still needs to be fully elucidated [29].

Despite the proliferation of scientific literature on the relationship between gut microbiota and neurodegenerative diseases [41,42,43,44,45], there remains a lack of metaproteomic, systematic, and rigorous investigations into this phenomenon. Few studies have utilized metaproteomic methods to explore the role of gut microbiota in the development and progression of Alzheimer’s disease. In response to this gap in the literature, the present study sought to address the issue by conducting a comprehensive analysis of the microbiota and proteome profiles in both young and old control models, as well as in early and late-onset AD models. By examining these various cohorts, the study aimed to elucidate the dynamic linkages between the gut microbiota and AD progression to identify potential therapeutic targets for this important disease. Ultimately, the findings of this study provide valuable insights into the complex interplay between gut microbiota and neuroinflammation in the context of AD, shedding new light on the pathophysiology of this disease and offering new avenues for therapeutic intervention.

## 2. Results

A cohort of 12 murine subjects was selected and sorted into two cohorts, the Tg and control groups. Each cohort was subsequently divided into biological samples collected at 3 and 12 months, with each sample replicated three times to ensure statistical rigor. The murine intestines were extracted and dissected, cryopreserved at −80 °C, and later thawed at room temperature to initiate experimentation. Upon examining the samples with nLC-MS/MS (Supplementary Figure S1), it was discerned that 2046 peptide sequences were observed in the 3-month control samples, 1540 peptide sequences were observed in the 3-month Tg samples, 2832 peptide sequences were observed in the 12-month control samples, and 2675 peptide sequences were observed in the 12-month Tg samples. Excluded microorganisms that did not fall within the three primary superkingdoms of life (Archaea, Bacteria, and Eukaryota) to ensure the validity and specificity of the results. Additionally, any sequences found to be identical or highly similar were removed from the unique sequences analyzed to avoid over-representation and redundancy (Table 1).

### 2.1. Metaproteomic Alterations in the Microbiota of Natural Aging

**Figure 1:**
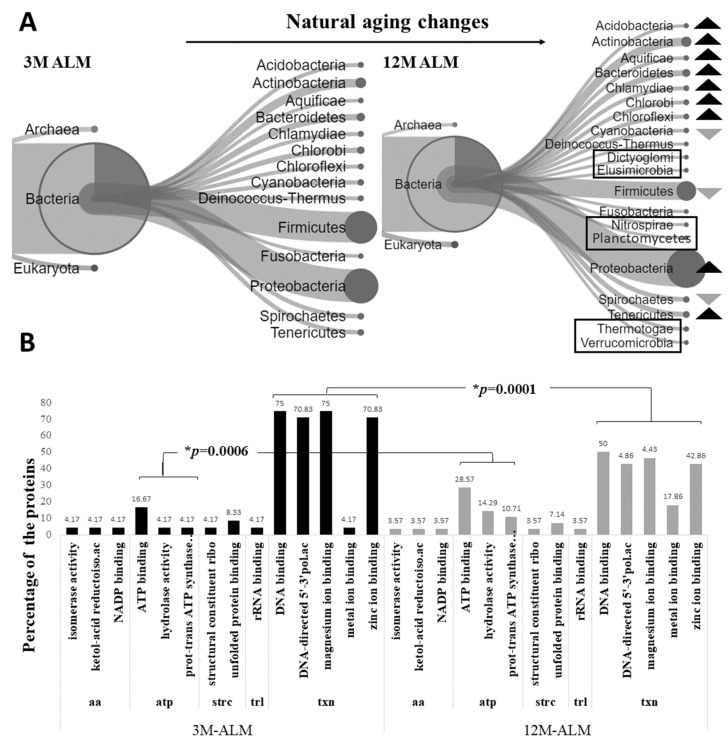
Examining the changes in the metaproteome of the microbiota associated with natural aging. The percentile comparison of the phyla diagram in (**A**) indicates an increase in certain phyla compared to the 3M-ALM, as denoted by the dark arrow rather than the light arrow. The highlighted taxa in the diagram represent unique phyla. The bar graph in (**B**) illustrates significant alterations in the molecular functions of the microbiota, as indicated by changes in protein expression; investigating the proteins and pathways associated with the common 214 bacteria co-expressed proteins in both 3M-ALM and 12M-ALM mice. The changes in transcription (*p* = 0.0001) and energy metabolism (*p* = 0.0006) functions are particularly noteworthy, with (*) *p*-values less than 0.05 indicating statistical significance. The protein pathways involved in these changes include transcription (txn), energy (atp), amino acid metabolism (aa), protein conformational structure (strc), and translation (trl). Unique phyla have been emphasized by shapes/boxes.

The Unipept implementation of the LCA algorithm was deemed appropriate for the study as the primary objective of this investigation was to draw a comparison between the microbiota of Tg and control mice. The unipept pept2lca command was utilized to apply this algorithm to control the cohort’s tryptic peptide. Specifically, we assessed the percentage change in the metaproteomic profile of normal aging microbiota by comparing 3m-ALM and 12m-ALM (Figure 1; Supplementary Figure S2). Following the Venn clustering method, we observed that the 3m-ALM and 12m-ALM flora contained 107 and 160 unique organisms, respectively, while 214 organisms were identified as homologous (Supplementary Figure S2A). During the natural aging process, the microbiota diversity of the late-stage flora was observed to increase compared to the early stage. This diversity increase was associated with the relative increase of nine phyla (*Acidobacteria*, *Actinobacteria*, *Aquificae*, *Bacteroidetes*, *Chlamydiae*, *Chlorobi*, *Chloroflexi*, *Tenericutes*, and *Proteobacteria*) compared to the preliminary period. However, three phyla, including *Cyanobacteria*, *Firmicutes*, and *Spirochaetes*, were observed to decrease relatively. Furthermore, unique phyla (*Elusimicrobi, Dictyoglomi*, *Nitrospirae*, *Planctomycetes*, *Thermotogae*, and *Verrucomicrobia*) were observed in the late stage. Due to the descriptive nature of this study, no preliminary data are available to ascertain the statistical significance of the observed alterations in the phyla. Nonetheless, the test for one proportion was applied using the MedCalc software, with the 3M-ALM values serving as a reference for the null hypothesis. Subsequently, four phyla, namely *Spirochaetes, Tenericutes, Proteobacteria*, and *Chlamydiae*, were deemed to undergo significant changes, as evidenced by the results of the proportion test (Figure 1A; Supplementary Figure S2B). Moreover, a comparative analysis of the phylum and proteome profiles demonstrated noteworthy variations that were statistically significant (Figure 1B). Once the alterations in the microbial flora during the natural aging process were analyzed, a *t*-test was conducted to investigate changes in the proteome profile. This was accomplished by identifying homologous-expressed proteins from the homologous organisms of the 3M-ALM and 12M-ALM flora. The homolog organisms numbered 214 (Supplementary Figure S2A), with 79 homologous proteins expressed by these organisms. Out of these, 46 proteins were found to be significantly expressed (*p* < 0.05) after the *t*-test, with a fold change (FC) and cut-off (0.25 < FC < −0.20) applied to the significant values. The resulting significant values yielded 20 decreased and 26 increased substantial values. Molecular function analysis was then performed on the homologous microbial sequences using *Unipept 4.3*, with the bar graph depicting the percentage of changing proteins (Figure 1B).

### 2.2. Metaproteomic Alterations in the Microbiota of Transgenic Aging

**Figure 2:**
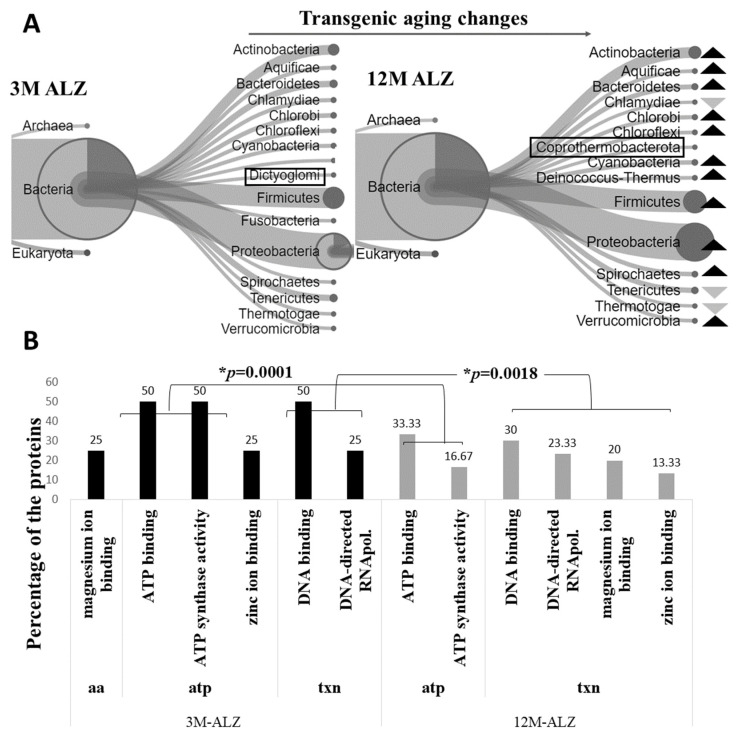
Representation of the changing patterns of the metaproteome in the Tg cohort microbiota. The percentile comparison of the phyla diagram (**A**) indicated a discernible increase in the dark arrow relative to the 3M-ALZ rather than the light arrow. Furthermore, the highlighted taxa in the diagram represented unique phyla. The bar graph (**B**) presented a significant alteration (* *p* < 0.05) in the molecular functions of the 159 homologous flora, particularly in the transcription (*p* = 0.0018) and energy metabolism (*p* = 0.0001) pathways. The molecular functions of the identified proteins were determined, and the differences were statistically evaluated using the MedCalc-Test for one proportion statistic. txn: transcription pathways, atp: energy pathways, aa: amino acid metabolism pathways. Unique phyla have been emphasized by shapes/boxes.

After scrutinizing the changes that occurred during the natural aging process, the study investigated the transformation of intestinal flora in Tg mice during aging. Likewise, the modifications that occurred in the intestinal flora, as well as the homologous expression of protein profiles, were examined in Tg mice aged between 3 and 12 months (3M-ALZ and 12M-ALZ, respectively). The phylum-based sequences were determined using the *Unipept* implementation of the LCA algorithm, and the aging process of these phyla in 3M-ALZ and 12M-ALZ cohorts were compared using both Venn diagrams and percentiles. In the microbial perspective of age-related diseases, the early stage displayed a unique set of 77 organisms, while this number increased to 189 in the later stage. The number of homologous organisms observed was 159 (Supplementary Figure S3A). Despite the absence of any significant alteration in the diversity of microbiota during the progression of the disease from its early to late stages, phyla demonstrate notable variations relative to each other. Specifically, 11 phyla, namely *Actinobacteria*, *Aquificae, Bacteroidetes, Chlorobi, Chloroflexi*, *Cyanobacteria*, *Deinococcus*, *Firmicutes*, *Proteobacteria*, *Spirochaetes*, and *Verrucomicrobia*, exhibit an increase, whereas three phyla, namely *Chlamydiae*, *Tenericutes*, and *Thermotoga*, manifest a decrease in abundance (Figure 2A). Furthermore, based on the applied statistical proportion test, the changes in five phyla, namely *Spirochaetes*, *Firmicutes*, *Proteobacteria*, *Cyanobacteria*, and *Actinobacteria*, are determined to be statistically significant (Supplementary Figure S3B). Even though the natural aging process exhibits predominantly stable changes and relatively small variations, the models representing the aging process of Tg cohorts display notable variability and instability. Additionally, the diversity observed in the natural aging process, where new organisms emerge, is not evident in the aging process of patients. Following the observation of microbial changes in the Tg aging process, the proteome profiles of the proteins homologously expressed from the corresponding phyla were analyzed, and their intensities were subjected to *t*-testing. The fold change over the average of the intensities was calculated to determine the significant increase and decrease of percentage values. Subsequently, the molecular functions in which the proteins of interest played a role were identified, and the values of these functions were compared using a proportion test. (Figure 2B).

### 2.3. Homolog Metaproteome Alterations in the Core Microbiota of 3M ALZ and 12M ALZ Cohorts

**Figure 3:**
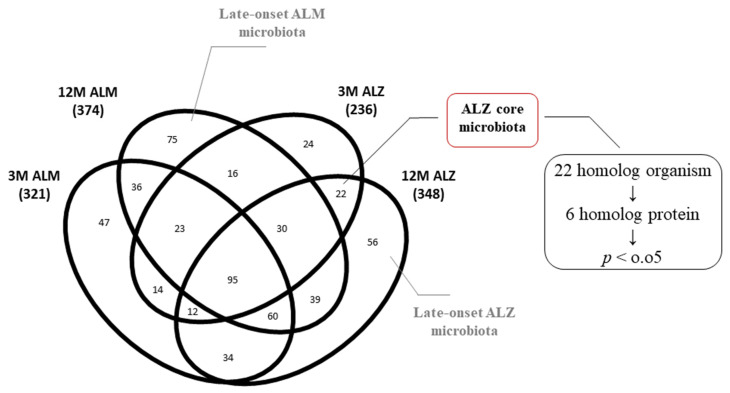
The analysis chart of the homolog 22 organisms. The Venn diagram emphasizes the presence of only 22 organisms that constitute the 3M ALZ and 12M ALZ core flora.

The present investigation endeavors to explore the ramifications of microbiota changes on the intestinal flora during the aging process, employing metaproteomic analysis. Specifically, we scrutinized the intestinal flora of a patient model suffering from AD, as well as of normal aging mice. Our research delved into the organisms and proteins observed in the homolog microbiota of 3M ALZ and 12M ALZ mice (22 bacteria), the microbiota of exclusively/pure 12M ALZ mice (56 bacteria), and the microbiota of solely/pure 12M ALM mice (75 bacteria). By comparing these diverse microbiotas, we could discern substantial differences in the organism and proteome composition of late-stage AD flora relative to that of normal-aged flora. Our investigation focused on scrutinizing the ALZ flora within the homolog organisms without discerning between the late and early stages of AD. We identified six homolog proteins that underwent significant alterations, whose relevance to AD was corroborated by existing literature. Upon subjecting these proteins to molecular functional analysis, we discovered their involvement in pathways associated with transcription and energy mechanism, akin to those found during aging. Furthermore, our investigation unearthed the identification of proteins linked with the penthose–phosphate path associated with oxidative stress, which was observed to be elevated in our study (Figure 3, Table 2 and Table 3). This inquiry facilitated our ability to appraise the core microbiota of the Tg model, irrespective of age, and reinforced the relationship between the phyla identified in the ALZ microbiota and AD, as previously substantiated in literature.

### 2.4. Metaproteomic Alterations in the 12M Microbiota of Pure ALZ and ALM Models

**Figure 4:**
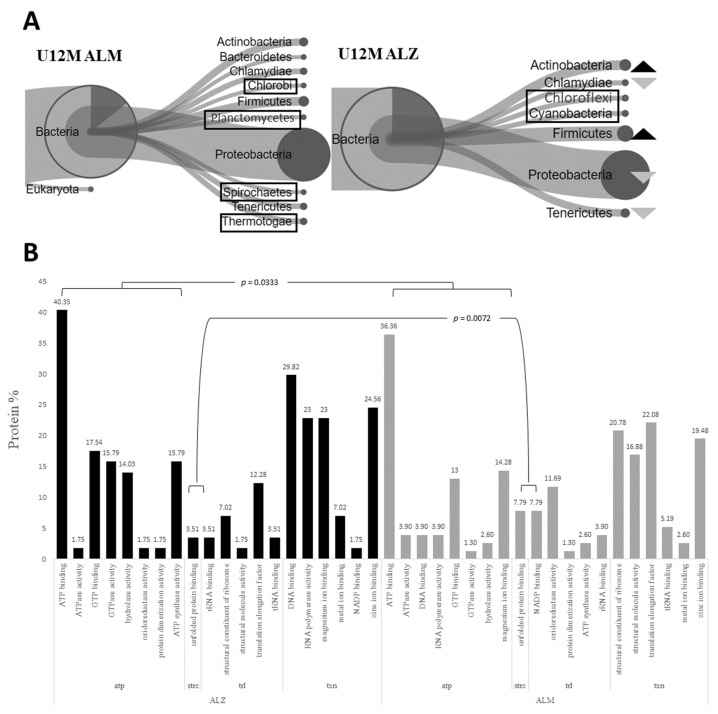
The metaproteomic changes observed in pure 12M ALZ and 12M ALM microbiota. (**A**) The percentage phylum diagram, which compares these organisms based on phylum. The dark arrow indicates an increase in ALZ compared to ALM, while the light arrows depict a decrease. The highlighted phyla indicate the unique phyla (U) of each biological sample. (**B**) The Test for one proportion statistic, which shows the statistical significance observed in the energy mechanism (*p* = 0.0333) and the pathways related to the protein 3-dimensional conformational structure (*p* = 0.0072). atp: energy metabolism, trl: translation pathways, txn: transcription pathways, strc: protein 3D conformational structure. Unique phyla have been emphasized by shapes/boxes.

In the ensuing phase, we embarked on the assessment of the discernible disparities in the microbiota and proteins of the pure 12-month ALZ and pure 12-month ALM from other models. Accordingly, we conducted a comparative analysis with the pure 12-month ALM to demarcate it from the regular aging data. The phylum diagram was first constructed to juxtapose the phyla percentages between the two cohorts. Our findings evinced a conspicuous upsurge in two phyla (Actinobacteria, Firmicutes) in contrast to the 12-month ALM flora and a corresponding decline in three phyla (Chlamydiae, Proteobacteria, Tenericutes) (Figure 4A). Furthermore, using the Test for one proportion statistic, we identified significant phyla (Proteobacteria: *p* < 0.0001), which are exhibited in the bar graph (Supplementary Figure S4). Conversely, owing to the presence of unique organisms in both microbiotas with no homolog organisms, it was not possible to conduct a molecular analysis of homolog proteins. Nevertheless, individual molecular analyses were conducted on the distinct proteins of each group (12M ALM → 56 organisms; 12M ALZ → 75 organisms). Consequently, homolog molecular functions were determined, and a noteworthy alteration was observed in the ALZ flora, as compared to the ALM flora in the pathways related to energy mechanism (*p* = 0.033) and protein 3D structure (*p* = 0.007) (Figure 4B).

## 3. Discussion

### 3.1. Metaproteomic Changes in the Microbiota of Natural Aging

Part of the investigation centers on the alterations in the microbiota that occur naturally during aging, with particular emphasis on the impact of the heightened abundance of certain phyla, specifically *Chlamydia*, *Proteobacteria*, and *Tenericutes*. Earlier inquiries have confirmed that an upsurge in the *Chlamydiae* phylum, particularly the *Chlamydia pneumonia* subspecies, exerts an unfavorable effect on the intestinal microbiota due to dysbiosis [46]. Moreover, *C. pneumonia* has been linked to immune suppression and Alzheimer’s Disease in the context of aging [47]. Studies have demonstrated that *C. pneumonia* can infect monocytes, as well as instigate inflammatory pathways and the formation of amyloid deposits in the brains of mice [48,49,50]. Meanwhile, although *Tenericutes* are usually observed in lower levels in natural microbiota [51], their increase may be indicative of dysbiosis. One investigation has revealed that the *M. gallisepticum* subspecies of *Tenericutes* can hinder the promoter activity of the mRNA–ribosome complex, thereby inducing significant modifications in the translatome in the presence of stressors [52]. Within the microbiota landscape, it is widely accepted that Proteobacteria reign supreme in their prevalence. However, a surfeit of this phylum, notably *Helicobacter, epsilon (Campylobacter)*, *Enterobacter*, and *Oligoflexia (Bdellovibria)* species, causes an intestinal microbiota dysbiosis that supports AD-linked dementia. Investigations featuring the 5xFAD model divulge that a magnification of Proteobacteria evokes inflammation along with bacterial endotoxins such as LPS, IL-8/10, and Aβ40,42. These toxins impede the gut microbiota and permeate particular brain regions via circulation. Furthermore, post-mortem studies reveal that the tally of *Proteobacteria* colonies in the brains of AD-afflicted individuals surpasses those of normal aging brains by a significant margin. Within this purview, the upsurge of the *Proteobacteria* phylum appears to be intimately associated with inflammaging, a chronic inflammatory state observed in the aging process. Significantly, a decrease in the *Spirochaetes* phylum, responsible for neurospirochetosis, occurs during natural aging. Evidence has substantiated the linkage between the *Spirochaetes* phylum and AD, eliciting atrophy and brain microgliosis [53,54,55]. Consequently, the reduction in *Spirochaetes* phylum, which is associated with chronic inflammation in AD, does not appear to be a causal factor in the development of AD. Therefore, this reduction may serve as a potential differentiating marker between AD and the microbiota of individuals undergoing normal aging. Hence, the absence of bacterial phyla that induces inflammation intimates that Tg aging is unlikely to arise from the chronic inflammatory pathway that usually characterizes dementia during natural aging. Regarding our observations, certain phyla could appear to serve a stabilizing function in combating persistent infections in the microbiota of models undergoing the natural aging process. In the literature, the *Acidobacteria* phylum has been observed to mitigate AD symptoms by mediating microglia-induced inflammation via metabolites [56,57]. In contrast, the reduction of the *Cyanobacteria* phylum, which is acknowledged for producing neurotoxins such as BMAA (β-N-methylamino-l-alanine), saxitoxin, and anatoxin-α, is associated with neurological diseases and an increased risk of AD. It is worth highlighting that *Cyanobacteria* is typically present in minute quantities in the core microbiota [58,59]. The unique presence or expansion of the *Verrucomicrobia* phylum is associated with inflammaging and AD [60]. Furthermore, the exclusive presence of the *Thermotogae* phylum [61] appears to elicit a beneficial effect on the AD flora, producing advantageous molecules like butyrate. The modest elevation of Actinobacteria, Bacteroidetes, and *Chloroflexi* phyla observed in the normal aging microbiota corroborates the theory of inflammaging. Dr. Fischer, who made the initial discovery of Alzheimer’s Disease alongside Alois A, identified *Actinobacteria* phylum as a critical mediator of inflammation through its ability to form bacterial colonies in the brain, a phenomenon strongly linked to AD [35,62,63,64,65]. While the *Bacteroidetes* phylum is present in the normal microbiota, research suggests that its overabundance may trigger an increase in lipopolysaccharides (LPS) [66,67]. Additionally, *Chloroflexi* [68,69,70,71,72,73,74] phylum is known to generate bacterial amyloid and is associated with AD. Finally, an absence of *Chlorobi*, *Dictyoglomi*, and *Nitrospirae* phyla in the AD-related literature, but their presence in our dataset suggests that these phyla may serve as valuable markers. Specifically, the *Chlorobi* phylum exhibits a slight increase in the late-stage Tg and control models. In addition, the conspicuous surge of the phylum in the early-stage Tg model suggests that it holds promise as an early-stage flora marker. Furthermore, the *Nitrospirae* phylum has been reported to escalate in cases of chronic dental inflammation, culminating in cognitive impairment [75]. Despite the low incidence rate of deleterious bacteria in normal aging, their close correlation with AD flora cannot be refuted. The presence of these bacteria in the 12M-ALM may shed light on the etiology of dementia in individuals who exhibit plaques in their brain but lack AD. It is postulated that these bacteria, which coexist with dementia-inducing ones and are labeled as stabilizers, may effectively impede chronic inflammation and, hence, prevent the onset of chronic dementia. Following the analysis of the microbial changes in the normal aging process, a *t*-test was conducted on the protein intensities by identifying homologous-expressed proteins from the homologous organisms of the 3M-ALM and 12M-ALM flora to explore changes in the proteome profile (Figure 1B). The analysis revealed significant differences in both transcription and energy metabolism functions, while the changing proteome profile was found to be correlated with changes in the phylum.

### 3.2. Metaproteomic Changes in the Microbiota of Transgenic (Tg) Aging

Despite relatively minor changes observed in natural aging, which are dominated by stabilization, models representing the aging process in Tg cohorts have become more variable and unstable. Moreover, the diversity observed in natural aging, with the presence of new organisms, has not been observed in Tg aging. The significant increase in inflammation-causing phyla, such as *Actinobacteria* [35,48,49], *Cyanobacteria* [39,50,51], *Firmicutes* [30,49,52,53,54,55,56,57], *Proteobacteria* [30,39,48,58,59,60], and *Spirochaetes* [61,62,63], as determined by the Test for one proportion statistics, lends support to the hypothesis that these phyla are closely connected to inflammation. Research studies have exemplified that an increase in *Cyanobacteria* leads to neurotoxicity [39], and that the *Firmicutes* phylum in the 5XFAD microbiota is associated with inflammation [30,64]. Furthermore, disproportionate increases in the *Proteobacteria* phylum, including gram-negative bacteria, have a linear relationship with IL-8/10 (Interleukins-8/10), LPS, and αβ 1-40/42 (amyloid-beta) [30,60]. The *Spirochaetes* phylum, associated with neurospirochaetesis [62], decreased significantly in natural aging but increased significantly in Tg aging, implying that this phylum could be regarded as a marker. Finally, it is worth noting that while the *Actinobacteria* phylum, characterized by inflammation [65], does not significantly expand in natural aging, it does significantly expand in Tg aging. Our discoveries proffer further corroboration for our hypothesis, as the phyla *Bacteroidetes*, *Chloroflexi*, and *Verrucomicrobia* establish an escalation, while the phyla *Fusobacteria, Tenericutes,* and *Thermotogae* exhibit a reduction. Notably, the conspicuous upgrade of *Bacteroidetes* [39] and *Chloroflexi* [53,66] in Tg mice is closely correlated to inflammation, while the prevalence of *Verrucomicrobia* [67,68], *Fusobacteria* [69,70], *Tenericutes* [71], and the butyrate source *Thermotogae* [72] phyla is noticeable in natural microbiota. Moreover, even though the association between the *Coprothermobacterota* phylum and AD has not been documented in the literature, our data “purely” demonstrates this phylum in the 3M-ALZ-versus-12M-ALZ and 12M-ALM-versus-12M-ALM models. Hence, this finding prompts us to contemplate operating the *Coprothermobacterota* phylum as a marker for late-onset microbiota. The observed microbial shifts in the Tg aging process have prompted a molecular investigation of the underlying mechanisms. We conducted a proteome analysis of the proteins expressed through homolog phyla, executing a *t*-test on their intensities (Table 2). Our functional analysis, guided by literature reviews, has revealed statistically momentous molecular changes, including amino acid metabolism, transcription pathways, and energy metabolism. Accordingly, dysregulated amino acid metabolism may serve as a hallmark mechanism in the Tg flora. In contrast to control models, the Tg aging process was denoted by a shift toward harmful bacteria and a reduction in beneficial bacteria. However, further research is necessary to probe the possible roles of the *Aquificae*, *Chlorobi*, *Deinococcus*–*Thermus* (unreported in the literature), and *Coprothermobacterota* phyla as markers in Tg aging. Our study underscores the demand for more comprehensive inquiries into these pathways to identify potential biomarkers and therapeutic targets. Altogether, our findings supply valuable perspicuity into the molecular mechanisms underlying microbial changes in Tg aging and accentuate the importance of understanding the intricate interplay between microbiota and host health.

### 3.3. Metaproteome Changing of Homolog 3M- and 12M-ALZ Core Microbiota

The aim of our endeavors for exploring the repercussions of microbiota shifts on intestinal flora during the aging process by employing metaproteomics analysis. Concretely, our scrutiny focuses on the core microbiota of Tg models, which disregards chronological age, dissecting extensively. The discoveries of our investigation put forth a strong correlation between the Tg model microbiota and the phyla found in AD, which has been well-substantiated by the existing literature. Our comprehensive examination reveals the presence of phyla *Actinobacteria* [35,48,49,65], *Firmicutes* [30,49,52,53,54,55,56,57], *Proteobacteria* [30,39,48,58,59,60], and *Bacteroidetes* [73,74], which are closely coupled with inflammatory responses. Moreover, we detected the presence of *Cyanobacteria* [39,50,51], comprehended for their neurotoxic impact, and *Tenericutes* [71], recognized for inflicting damage to the mRNA–ribosome complex. Within this cohort, we identified six homolog proteins that exhibited considerable differences in the proteins they express, which have been documented in the existing literature. Prior investigations into ATP synthase [75,76,77,78] have illustrated that impaired mitochondrial oxidative phosphorylation can trigger oxidative stress, which, in turn, interrupts energy production. Meanwhile, the 60-kDa chaperonin [79,80,81,82] protein has been demonstrated to possess anti-stress properties and safeguard neurons from degeneration, particularly in response to amyloid toxicity. The noteworthy increase in this protein observed in our data implies a parallel with the upregulation of chaperones in a dysbiotic flora characterized by chronic inflammation and oxidative stress. Transaldolase protein [83,84,85] is crucial in maintaining metabolite balance in the pentose–phosphate pathway. Studies have revealed that it is induced during oxidative stress and classified as a functional category in drosophila with cognitive impairment, aligning with the significant increase in this protein we observed. Additionally, our available analysis found that pentose–phosphate pathways are impacted in the transcription and energy metabolism axis. However, earlier research on linker histone–DNA complexes in Alzheimer’s Disease has identified DNA-binding proteins [86,87] as critical factors in regulating genetic processes like transcription. Our data showed a noteworthy increase and decrease in transcription pathways correlating with a significant reduction in DNA-binding proteins, suggesting that a certain range of increases and decreases in this protein negatively affects transcription.

### 3.4. Metaproteome Changing of Unique 12-Months ALZ and ALM Microbiota

We have comprehensively scrutinized microbiota differences during aging, employing an analysis of the core microbiota of transgenic aging models, focusing solely on the 12M-ALM and 12M-ALZ organisms and proteins filtered from other models. Our study unveiled a remarkable rise in the *Actinobacteria* phylum [35,48,49,65] and the *Firmicutes* phylum [30,49,52,53,54,55,56,57] in these representatives, which is consistent with the previous literature. Intriguingly, our results exhibit a noteworthy decline in the *Proteobacteria* phylum compared to other models. Our observations imply that these essential phyla recreate a balancing role in the flora, and perturbations in their abundance within specific ranges could cause neurodegeneration through dysbiosis and inflammation. Furthermore, we noticed the exclusive presence of the *Cyanobacteria* phylum [39,50,51], comprehended for its deleterious effects on the flora, solely in the pure 12-ALZ microbiota in our dataset, hinting at its possible usage as a biomarker for late-stage ALZ microbiota modeling. Additionally, we observed an augmentation in the *Chloroflexi* phylum in our data in late-stage Tg models and its distinctive presence in the early-stage ALZ model and the pure 12-ALZ microbiota, signifying its potential application as a biomarker in the early and late-stage ALZ, respectively.

**Figure 5:**
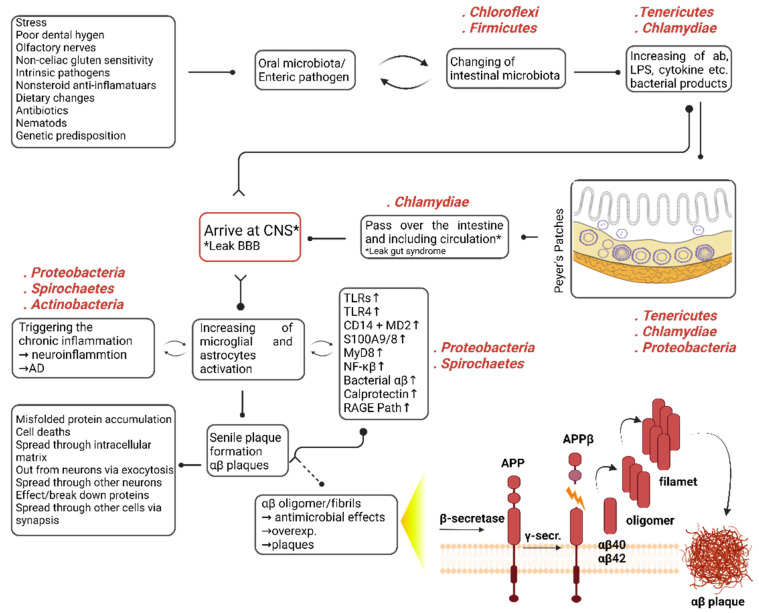
Exploring the hypothetical framework of gut–brain axis dysregulation in Alzheimer’s Disease: Insights derived from our empirical findings.

In summary, our postulation asserts that the mechanism of infection is initiated through a multifaceted interplay of endogenous and exogenous factors (Figure 5). The observed determinations are conjectured to be attributed to internal factors such as genetic predisposition [88,89], as well as extrinsic factors [90] encompassing the dysbiosis of the oral microbiome, exposure to stress or antibiotics, non-celiac gluten sensitivity, an injudicious diet, and enteric pathogens, which can instigate nematode proliferation. These factors can render gradual modifications in the microbiota, eventually conducting a chronic intestinal microbiota exchange. This exchange can culminate in an unwarranted escalation of bacterial products, such as intestinal amyloid, LPSs, and cytokines. These products can trigger chronic intestine inflammation, particularly through Peyer’s patches [90], which can evoke augmented intestinal permeability, Leaky Gut Syndrome. Bacteria or their products can then permeate and penetrate the CNS. Due to the intricate interplay between endogenous and exogenous factors and the bidirectional communication between the brain and gut that ensues with aging, these bacteria or their secondary metabolites can subsequently traverse the blood–brain barrier, which is commonly referred to as “Leaky BBB”. Typically, activating defensive cells such as microglia and astrocytes by bacteria or their products in the brain can result in acute inflammation. Consequently, the amyloid precursor protein (APP) extends beta-amyloid (aβ) oligomers that exhibit antimicrobial functions through TNF-α and its converting enzyme. These oligomers evolve activated through oligomerization, a natural process [7]. Nonetheless, in the context of advanced age, which tends chronic inflammation, genetic predisposition [91], or the silent period in newborns [45], a self-propagating cycle between the bacterial density and their products and defensive cells such as macrophages and astrocytes can emerge. In situations of chronic inflammation, the activation of molecules or pathways such as TLRs, TLR4, CD14 + MD2, S100A9/8, MyD8, NF-κB, Bacterial Aβ, Calprotectin, and RAGE Path is likely to materialize [92,93,94,95,96,97]. Under such circumstances, an excessive antimicrobial response from aβ is anticipated to usher in plaque formation. This is due to the chronic antimicrobial response, which results in the accumulation of misfolded proteins, cell death, distribution from the intracellular matrix to the extracellular matrix, and diffusion to other cells, disrupting protein structure [7,98]. When evolved proteins are dysfunctional or malfunction, the equilibrium within the system is perturbed. Our analysis, which we attempted with various permutations, consistently indicated that microbial dysbiosis of the flora primarily impacts amino acid, energy metabolism, and transcription pathways. The statistically significant alterations in the expression of these molecular pathways may signify the system’s response or the beneficial bacteria to restore homeostasis in the face of microbial dysbiosis. Despite the decrease in protein expressions in various molecular functions, the concomitant boost in proteins responsible for identical operations implies that the flora may strive to maintain stability and avoid disrupting the balance.

Replication and further analyses are warranted to validate the findings of our study. Nonetheless, valuable inferences have been derived both from previous studies and this investigation (Table 4).

Drawing insights from our data, we propose that the observed increases in Cyanobacteria and Firmicutes phyla could potentially serve as late-stage markers for Alzheimer’s Disease (AD). Conversely, the declines in Chlamydiae and Tenericutes phyla during late-stage AD imply their potential beneficial role, thereby suggesting their absence as indicative late-stage markers. Moreover, the augmented abundance and presence of Chloroflexi and Actinobacteria phyla during late-stage AD raise intriguing possibilities of considering them as markers of the aging process. Notably, Proteobacteria phylum exhibited both increases and decreases across all examined groups. This dichotomous behavior suggests that deviations from an optimal range may have detrimental effects on the microbiota. Consequently, alterations in the Proteobacteria phylum beyond a certain threshold could be regarded as potential markers of disease progression. In addition, the presence of Spirochaetes and the unique presence of Coprothermobacterota within the 3-12M AD flora warrant focused investigations to ascertain their potential role as harmful-marker bacteria within the natural flora. Conversely, the presence of Tenericutes and the exclusive presence of Thermotogae in the 3-12M ALM flora beckon further exploration into their potential as beneficial-marker bacteria for the natural flora. In our study, a meticulous examination of molecular functional profiles across all biological samples revealed notable and significant transformations in the transcription and energy metabolism pathways within the microbiota of the Tg model. This noteworthy discovery prompts speculation regarding the potential role of these pathways as molecular-level biochemical markers. Intriguingly, our data also unveil a compelling association between the abundance of certain proteins, such as ATP synthase, 60 kDa chaperonin (GroEL protein), DNA-directed RNA polymerase, and Transaldolase, expressed by dominant phyla in the AD microbiota and the observed alterations in the transcription and energy metabolism pathways.

## 4. Materials and Methods

### 4.1. Murine Samples

The experimental design involved the use of 5XFAD transgenic mice and their littermates that carry mutations of three familial Alzheimer’s Disease-associated genes and two human presenilin genes (Jackson Laboratory, Bar Harbor, Maine, ABD). Stool samples were classified into two distinct biological groups based on transgene expression, namely Tg and control. Each group was then further divided into four separate biological samples, representing both early and late stages of the disease. Moreover, three biological replicates were prepared for each biological sample, ensuring robustness and reproducibility of the results.

### 4.2. Microbial Extraction from Murine Fecal Samples

The murine intestinal tissues obtained by dissection were stored at −80 °C until utilized in the experiment. Upon thawing at room temperature, the samples underwent centrifugation. A methodology previously described by researchers [99,100] was employed to isolate bacterial samples from fecal matter. In brief, the samples were vortexed with 10 mL of phosphate-buffered saline (PBS), followed by shaking the samples on a rotator for 45 min. This process was repeated thrice with centrifugation at 500× *g* to obtain the supernatant. Subsequently, the supernatant was subjected to centrifugation at 20,000× *g* for 15 min. Twenty-five milligrams were weighed and subjected to protein extraction from the resultant pellets.

### 4.3. Protein Extraction

The pellets of 25 mg were supplemented with the extraction buffer (2% SDS, 100 mM DTT, 20 mM Tris-HCl, pH 8.8) and were subjected to an incubation period of 20 min at 95 °C while being shaken at 500 rpm (TS-100, Thermo–Shaker, Biosan SIA, Ratsupites iela7k-2, Riga, Latvia, LV-1067). Next, the samples were frozen for 10 min at −80 °C, followed by heating at 95 °C for additional 10 min. The samples were then sonicated for 10 s (Hielsher UP200St Vialtweeter, Teltow, Germany) and centrifuged at 20,000× *g* for 10 min. The supernatant was collected and stored at −80 °C for subsequent analysis. The results are verified by SDS–PAGE analysis by using the TGX Stain-Free™ FastCast™ Acrylamide Solutions kit (Bio-Rad Laboratories, TNC, Hercules, CA, USA Catalog. NO. 161–0181 as per the established literature [101]. In brief, 0.75-mm glass plates were utilized; Resolver A and B were prepared as 2 mL, and Stacker A and B were 1 mL, respectively. The gel was poured into the cassette, and electrophoresis was performed at 70 V for the first 15 min, followed by 150 V. The resulting gels were subjected to three washes with ultrapure water (Milli Q) using a Benchmark Scientific Blot Boy Fixed Speed 3D laboratory shaker. Finally, the gels were stained with Coomassie Brilliant Blue R-250 Dye (20278) and visualized using the ChemiDocTM MP System (BioRad, Hercules, CA, USA).

### 4.4. Microdialysis and Protein Concentration Determination

The lysates were subjected to dialysis against 20 mM Ammonium Bicarbonate, pH 7.8, overnight at 4 °C using the Slide-A-Lyzer ™ MINI Dialysis Unit (Thermo Fisher Scientific, Rockford, IL, USA). Following dialysis, the samples were transferred to LoBind^®^ tubes. The concentration of proteins in the samples was determined using the Micro BCA ™ Protein Assay Kit. Measurements were obtained using the Synergy ™ HTX Multi-Mode Microplate Reader (BioTek Instruments, Winooski, VT, USA).

### 4.5. FASP (Filter Aided Sample Preparation) Protocol

The FASP process was initiated by adapting the protocol provided by the commercial kit, combined with the protocols described by Wisniewski et al. (2016) [102]. Briefly, 100 µg of protein lysate were loaded onto 30-kDa cut-off spin columns and centrifuged at 14,000× *g* for 15 min after dissolving the urea in 8M Tris-HCL, resulting in a final volume of 230 µL. The samples were thoroughly washed with 200 µL of urea buffer and treated with 10 mM of DTT (Pierce ™ DDT, Dithiothreitol, 20290) for 30 min at 500 rpm and 56 °C. Immediately after that, the samples were alkylated in the dark for 20 min using Iodoacetamide (IAA BioUltra, 1149). Subsequently, the samples were washed thrice with urea buffer, followed by three washes with 50-mM ammonium bicarbonate. After that, 3.3 µg of trypsin were added to each sample and incubated for 18 h at 37 °C. The next day, peptide mixtures were obtained by washing the filters with 50 µL of elution buffer (Acetonitrile (ACN): Water: Formic Acid; 7:2:1). The tubes were exposed to vacuum at room temperature for 20 min to remove ACN using the Vacufuge Vacuum Concentrators (Eppendorf). The peptide concentration was determined using a Quantitative Colorimetric Peptide Assay (Pierce™). Each sample was prepared at 200 ng/µL, and 5 µL of the sample were used for each LC-MS/MS analysis using the Xevo G2-XS QTof Quad Flight Time Mass Spectrometer. The remaining samples were stored at −80 °C.

### 4.6. Nano LC/MS-MS

The nLC-MS/MS analysis was conducted employing a cutting-edge ACQUITY UPLC M-Class coupled to a state-of-the-art SYNAPT Xevo G2-XS system (Waters). The column temperature was adjusted to 55 °C. The initial introduction of peptides onto a trap column (Symmetry C18 5 m, 180 m i.d. × 20 mm) was followed by their separation via gradient elution through an analytic column (CSH C18, 1.7 m, 75 m i.d. × 250 mm). A lock mass reference, Glu-1-fibrinopeptide B at a concentration of 100 fmol/µL, was utilized. The device was operated in positive ion mode. A novel data-independent mode of acquisition, named SONAR, was adopted for MS data collection with a 24-Da quadrupole transmission width. In insensitivity mode, without any precursor ion preselection, all ions in the 50–1950 m/z range were fragmented collectively.

### 4.7. Data Reduction and Analysis

Progenesis-QIP. The analytical framework employed in this study for data processing and analysis is Progenesis-QI for proteomics software (V.2.0 Waters). The database used for the analysis of the murine proteome was the Uniprot database. Peak intensity thresholds were set at 60 and 10 counts for low and elevated energy, respectively. The mass spectrometry data were analyzed with the following settings: minimum number of fragmented ion matches per peptide = 2, minimum number of fragment ion matches per protein = 5, minimum number of unique peptides per protein = 1, and maximum number of missed cleavage for tryptic digestion = 1. Carbamidomethyl C was selected as the fixed modification, while oxidation M and deamidation N and Q were chosen as variable modifications. False discovery rate (FDR) ≤ 1% was applied, and 1+ charged ions were eliminated. Normalization of the data between samples was achieved by utilizing the total ion intensity. The statistical program integrated into Progenesis-QI for proteomics software was employed to calculate expressional changes, as well as *p* and q values. Proteins were considered differentially expressed only if they met the following criteria: ANOVA *p* < 0.05, q < 0.05, unique peptide > 2, and fold change ≥ 1.3.

Unipept. Utilized the open-source web application, Unipept, to conduct a comprehensive analysis of the biodiversity and functional characteristics of our vast and intricate metaproteome samples [103]. Unipept’s interactive data visualizations allowed us to explore the complex relationships within our data effectively. Specifically, the application effectively indexed tryptic and sorted peptides, which were then retrieved from UniProt entries, facilitating the rapid retrieval of all occurrences of a tryptic peptide. The Unipept framework’s LCA algorithm was deployed to scrutinize the tryptic peptides of each cohort. The Unipept pept2lca command was utilized to enzymatically cleave the input peptide list into perfect tryptic peptides and compute their respective LCA. Unipept curated tryptic peptides acquired from UniProt sequences that are between 5 and 50 amino acids in length, inclusively. Thus, to determine the LCAs of each cohort’s peptides, the subsequent processing steps were executed: (i) Missed cleavages were subjected to further digestion; (ii) Peptides of insufficient or excessive length were excluded; and (iii) Resulting tryptic peptides were subjected to deduplication. The ultimate index of tryptic peptides was then passed onto the Unipept pept2lca command with the option to homogenize leucine and isoleucine. Accordingly, the prot2pept command has been utilized to transform protein sequences into peptides, and these peptides have been filtered using the peptfilter command. The resultant set of unique peptides has been sorted and counted via the sort -u|wc -l commands. Concerning the 3M-ALM group, the peptides and their corresponding taxonomic data have been archived in the “cohort_3M_ALM.csv” file, generated using the unipept pept2lca command that includes an option to equate leucine and isoleucine as mentioned above in detail. The time taken to execute the code for this cohort has also been displayed, indicating that the process lasted for 0.329 s in real time, 0.465 s in user mode, and 0.038 s in system mode.

### 4.8. Data Statistics

The test for one proportion in MedCalc has been used to assess whether the proportion of microbiota diversity in Tg/control sample significantly differs from control/Tg sample’ proportion for each cohort design. In MedCalc, the test statistic (z) is computed using the sample proportion (p), sample size (n), and hypothesized or known population proportion (P0) according to the formula z = (p − P0)/√(P0 ∗ (1 − P0)/n). The resulting z value is compared to a standard normal distribution table or calculated *p*-value using MedCalc’s built-in functions. The *p*-value is then compared to the chosen significance level (alpha) to determine whether to reject the null hypothesis (which assumes the population proportion is equal to P0) in favor of the alternative hypothesis (which posits that the population proportion is not equal to P0).

Limitations. This study exhibits two primary limitations. Firstly, the number of animals utilized in the research is relatively small, which may impact the statistical power and generalizability of the findings. Secondly, the validation of the study results could not be performed using alternative biochemical and computational techniques, apart from the nLC-MS/MS technique and Unipept suite.

In an academic context, it is important to present limitations in a clear and objective manner, highlighting potential areas where the study could have been strengthened or improved. By acknowledging these limitations, authors demonstrate a critical understanding of their work and provide insights into potential avenues for future research.

## 5. Conclusions

AD is a devastating form of dementia concerning millions worldwide. Despite substantial research on the relations between gut microbiota and neurodegeneration, the metaproteomic effects of microbiota on AD pathogenesis remain largely unexplored. This study aims to encounter the role of gut microbiota in AD, particularly concerning changes in the proteome and molecular pathways connected to disease progression. Using nLC-MS/MS, we compared 3M-ALZ and 3M-ALM mice (early AD) with 12M-ALZ and 12M-ALM mice (late-stage AD). Computational analysis disclosed valuable wisdom into the gut–brain axis, revealing that microbiota, proteome, and molecular modifications in the intestine occur well before disease symptoms. Discrepancies between normal-aged flora and late-stage AD mice propose potential markers for early and late AD stages. While acknowledging the need for a more comprehensive analysis of these phyla, pathways, and proteins, which we tentatively propose as putative markers, our pioneering metaproteomics-based pilot study contributes significantly to the existing literature, presenting a novel avenue for exploration and expanding our understanding of AD-associated microbial dynamics.

## Figures and Tables

**Table 1:** Data processing result of the study. The distribution of peptide, protein, and organism amounts across the biological samples analyzed. * Unique phyla.

	3M-ALM	12M-ALM	3M-ALZ	12MALZ
Peptide	502	636	338	584
Protein	484	636	338	584
Organism	321	374	236	348
Unique phyla	1 *	5 *	1 *	1 *
Phyla	15	19	16	16
	Acidobacteria Actinobacteria Aquificae Bacteroidetes Chlamydiae Chlorobi Chloroflexi Cyanobacteria Deinococcus-thermus * Elusimicrobia Firmicutes Fusobacteria Proteobacteria Spirochaetes Tenericutes	Acidobacteria Actinobacteria Aquificae Bacteroidetes Chlamydiae Chlorobi Chloroflexi Cyanobacteria Deinococcus-thermus * Dictiyoglomi Firmicutes Fusobacteria * Nitrospirae * Planctomycetes Proteobacteria Spirochaetes Tenericutes * Thermotogae * Verrumicrobia	Actinobacteria Aquificae Bacteroidetes Chlamydiae Chlorobi Chloroflexi Cyanobacteria Deinococcus-thermus * Dictiyoglomi Firmicutes Fusobacteria Proteobacteria Spirochaetes Tenericutes Thermotogae Verrumicrobia	Actinobacteria Aquificae Bacteroidetes Chlamydiae Chlorobi Chloroflexi * Coprothermobacterota Cyanobacteria Deinococcus-thermus Firmicutes Fusobacteria Proteobacteria Spirochaetes Tenericutes Thermotogae Verrumicrobia

**Table 2:** Identifying the most prominent homolog six proteins: an examination of Figure 4.

UniProt ID	Protein Name	*t*-Test	F-Chance	Organism	Phylum
P13357	ATP synthase subunit beta	0.01	−0.88	Cellulophaga lytica	Bacteroidetes
Q9XXK1	ATP synthase subunit alpha, mitochondrial	0.01	−0.08	Caenorhabditis elegans	Metazoa
P37282	60 kDa chaperonin	0.08	0.49	Lactococcus lactis subsp. lactis (strain IL1403)	Firmicutes
C0QVC8	Probable transaldolase	0.01	1.91	Brachyspira hyodysenteriae (strain ATCC 49526/WA1)	Spirochaetes
A0K2Y1	ATP synthase subunit alpha	0.01	9.31	Burkholderia cenocepacia (strain HI2424)	Proteobacteria
A8GV24	DNA-directed RNA polymerase subunit beta	0	11.62	Rickettsia bellii (strain OSU 85-389)	Proteobacteria

**Table 3:** Feature of the molecular functional analysis of the six homolog proteins that underwent significant changes, and these proteins were analyzed based on their peptide sequences: a close examination of Figure 4 and Table 2.

Pathways	Molecular Function	%Peptide
Energy metabolism	ATP binding	66.67
	Proton-transporting ATP synthase activity, rotational mechanism	50
	hydrolase activity	16.67
Pentose-phosphate	Sedoheptulose-7-phosphate: D-glyceraldehyde-3-phosphate glyceronetransferase activity	16.67
	Aldehyde-lyase activity	16.67
Protein structure	Unfolded protein binding	16.67
Transcription	DNA binding	16.67
	DNA-directed 5′-3′ RNA polymerase activity	16.67
	Ribonucleoside binding	16.67

**Table 4:** Speculative assessment of phylum variations in Alzheimer’s Disease microbiota based on our data. The presence of a plus sign denotes an observed increase, a minus sign indicates a decrease, the letter ‘u’ signifies the unique presence of a phylum within a specific group, and zero denotes the absence of a phylum within that group. Notably, the highlighted lines explicate the underlying rationales for considering these specific phyla as potential diagnostic markers. The cohorts examined include 3-12M ALM, representing the natural aging group; 3-12M ALZ, representing the transgenic aging groups; and U 12M ALZ, representing the exclusive 12-month Alzheimer’s disease cohort.

Phyla	3-12M ALM	3-12M ALZ	U 12M ALZ	Notes
Acidobacter	+	0	0	Prospective (beneficial)
Actinobacteria	+	+	+	Prospective (aging marker)
Aquificae	+	+	0	In progress
Bacteroidetes	+	+	0	In progress
Chlamydiae	+	−	−	Prospective (beneficial)
Chlorobi	+	+	0	In progress
Chloroflexi	+	+	u	Prospective (aging marker)
Cyanobacteria	−	+	u	Prospective (late-onset marker)
Deinococcus-Thermus	0	+	0	In progress
Dictyoglomi	u	0	0	For the first time
Firmicutes	−	+	+	Prospective (late-onset marker)
Fusobacteria	0	−	0	In progress
Nitrospirae	u	0	0	For the first time
Planctomycetes	u	0	0	For the first time
Proteobacteria	+	+	−	In progress
Spirochaetes	−	+	0	Prospective (harmful)
Tenericutes	+	−	−	Prospective (beneficial)
Thermotogae	u	−	0	Prospective (beneficial)
Verrucomicrobia	u	+	0	In progress
Coprothermobacterota	0	u	0	Prospective (harmful)

## Data Availability

The data presented in this study are available on request from the corresponding author. The data are not publicly available.

## Supplementary Materials

The following supporting information can be downloaded at: https://www.mdpi.com/article/10.3390/ijms241612819/s1.

## References

[1] Alagöz A.N. (2017). Mikrobiyota ve Nörodejenerasyon. J. Biotechnol. Strateg. Health Res..

[2] Xu Z., Knight R. (2014). Dietary effects on human gut microbiome diversity. Br. J. Nutr..

[3] Yılmaz K., Altındiş M. (2017). Sindirim sistemi mikrobiyotasi ve fekal transplantasyon. Nobel Med..

[4] Zhu X., Han Y., Du J., Liu R., Jin K., Yi W. (2017). Microbiota-gut-brain axis and the central nervous system. Oncotarget.

[5] Eckburg P.B., Bik E.M., Bernstein C.N., Purdom E., Dethlefsen L., Sargent M., Gill S.R., Nelson K.E., Relman D.A. (2005). Microbiology: Diversity of the human intestinal microbial flora. Science.

[6] Mandal R.S., Saha S., Das S. (2015). Metagenomic Surveys of Gut Microbiota. Genom. Proteom. Bioinform..

[7] Kowalski K., Mulak A. (2019). Brain-Gut-Microbiota Axis in Alzheimer’s Disease. J. Neurogastroenterol. Motil..

[8] Dinan T.G., Cryan J.F. (2016). Gut instincts: Microbiota as a key regulator of brain development, ageing and neurodegeneration. J. Physiol..

[9] Quigley E.M.M. (2017). Microbiota-Brain-Gut Axis and Neurodegenerative Diseases. Curr. Neurol. Neurosci. Rep..

[10] Westfall S., Lomis N., Kahouli I., Dia S.Y., Singh S.P., Prakash S. (2017). Microbiome, probiotics and neurodegenerative diseases: Deciphering the gut brain axis. Cell. Mol. Life Sci..

[11] Catanzaro R., Anzalone M., Calabrese F., Milazzo M., Capuana M., Italia A., Occhipinti S., Marotta F. (2014). The gut microbiota and its correlations with the central nervous system disorders. Panminerva Medica.

[12] Frasca D., Blomberg B.B. (2015). Inflammaging decreases adaptive and innate immune responses in mice and humans. Biogerontology.

[13] Köhler C., Maes M., Slyepchenko A., Berk M., Solmi M., Lanctôt K.L., Carvalho A.F. (2016). The Gut-Brain Axis, Including the Microbiome, Leaky Gut and Bacterial Translocation: Mechanisms and Pathophysiological Role in Alzheimer’s Disease. Curr. Pharm. Des..

[14] Cryan J.F., O’Riordan K.J., Cowan C.S., Sandhu K.V., Bastiaanssen T.F., Boehme M., Codagnone M.G., Cussotto S., Fulling C., Golubeva A.V. (2019). The microbiota-gut-brain axis. Physiol. Rev..

[15] Louveau A., Smirnov I., Keyes T.J., Eccles J.D., Rouhani S.J., Peske J.D., Derecki N.C., Castle D., Mandell J.W., Lee K.S. (2015). Structural and functional features of central nervous system lymphatic vessels. Nature.

[16] Yano J.M., Yu K., Donaldson G.P., Shastri G.G., Ann P., Ma L., Nagler C.R., Ismagilov R.F., Mazmanian S.K., Hsiao E.Y. (2015). Indigenous Bacteria from the Gut Microbiota Regulate Host Serotonin Biosynthesis. Cell.

[17] Bertrand P.P., Bertrand R.L. (2010). Serotonin release and uptake in the gastrointestinal tract. Auton. Neurosci..

[18] Bhattacharjee S., Lukiw W.J. (2013). Alzheimer’s disease and the microbiome. Front. Cell. Neurosci..

[19] Marizzoni M., Provasi S., Cattaneo A., Frisoni G.B. (2017). Microbiota and neurodegenerative diseases. Curr. Opin. Neurol..

[20] Tremlett H., Bauer K.C., Appel-Cresswell S., Finlay B.B., Waubant E. (2017). The gut microbiome in human neurological disease: A review. Ann. Neurol..

[21] Thal D.R., Rüb U., Orantes M., Braak H. (2002). Phases of Aβ-deposition in the human brain and its relevance for the devel-opment of AD. Neurology.

[22] Bayer, Bayer T.A., Wirths O. (2010). Intracellular accumulation of amyloid-beta—A predictor for synaptic dysfunction and neuron loss in Alzheimer’s disease. Front. Aging Neurosci..

[23] Oakley H., Cole S.L., Logan S., Maus E., Shao P., Craft J., Guillozet-Bongaarts A., Ohno M., Disterhoft J., Van Eldik L. (2006). Intraneuronal β-amyloid aggregates, neurodegeneration, and neuron loss in transgenic mice with five familial Alzheimer’s disease mutations: Potential factors in amyloid plaque formation. J. Neurosci..

[24] Girard S.D., Jacquet M., Baranger K., Migliorati M., Escoffier G., Bernard A., Khrestchatisky M., Féron F., Rivera S., Roman F.S. (2014). Onset of hippocampus-dependent memory impairments in 5XFAD transgenic mouse model of Alz-heimer’s disease. Hippocampus.

[25] Girard S.D., Baranger K., Gauthier C., Jacquet M., Bernard A., Escoffier G., Marchetti E., Khrestchatisky M., Rivera S., Roman F.S. (2013). Evidence for early cognitive impairment related to frontal cortex in the 5XFAD mouse model of Alz-heimer’s disease. J. Alzheimer’s Dis..

[26] Eimer W.A., Vassar R. (2013). Neuron loss in the 5XFAD mouse model of Alzheimer’s disease correlates with intraneuronal Aβ42 accumulation and Caspase-3 activation. Mol. Neurodegener..

[27] Jawhar S., Trawicka A., Jenneckens C., Bayer T.A., Wirths O. (2012). Motor deficits, neuron loss, and reduced anxiety coin-ciding with axonal degeneration and intraneuronal Aβ aggregation in the 5XFAD mouse model of Alzheimer’s disease. Neurobiol. Aging.

[28] Brandscheid C., Schuck F., Reinhardt S., Schäfer K.-H., Pietrzik C.U., Grimm M., Hartmann T., Schwiertz A., Endres K. (2017). Altered Gut Microbiome Composition and Tryptic Activity of the 5xFAD Alzheimer’s Mouse Model. J. Alzheimer’s Dis..

[29] Wang X., Sun G., Feng T., Zhang J., Huang X., Wang T., Geng M., Xie Z., Chu X., Yang J. (2019). Sodium oligomannate therapeutically remodels gut microbiota and suppresses gut bacterial amino ac-ids-shaped neuroinflammation to inhibit Alzheimer’s disease progression. Cell Res..

[30] Lee H.-J., Lee K.-E., Kim J.-K., Kim D.-H. (2019). Suppression of gut dysbiosis by Bifidobacterium longum alleviates cognitive decline in 5XFAD transgenic and aged mice. Sci. Rep..

[31] Cummings J., Lee G., Ritter A., Zhong K. (2018). Alzheimer’s disease drug development pipeline: 2018. Alzheimer’s Dement. Transl. Res. Clin. Interv..

[32] Kodamullil A.T., Zekri F., Sood M., Hengerer B., Canard L., McHale D., Hofmann-Apitius M. (2017). Trial watch: Tracing investment in drug development for Alzheimer disease. Nat. Rev. Drug Discov..

[33] Cattaneo A., Cattane N., Galluzzi S., Provasi S., Lopizzo N., Festari C., Ferrari C., Guerra U.P., Paghera B., Muscio C. (2017). Association of brain amyloidosis with pro-inflammatory gut bacterial taxa and peripheral inflammation markers in cognitively impaired elderly. Neurobiol. Aging.

[34] Vogt N.M., Kerby R.L., Dill-McFarland K.A., Harding S.J., Merluzzi A.P., Johnson S.C., Carlsson C.M., Asthana S., Zetterberg H., Blennow K. (2017). Gut microbiome alterations in Alzheimer’s disease. Sci. Rep..

[35] Zhuang Z.-Q., Shen L.-L., Li W.-W., Fu X., Zeng F., Gui L., Lü Y., Cai M., Zhu C., Tan Y.-L. (2018). Gut Microbiota is Altered in Patients with Alzheimer’s Disease. J. Alzheimer’s Dis..

[36] Calvani R., Picca A., Lo Monaco M.R., Landi F., Bernabei R., Marzetti E. (2018). Of Microbes and Minds: A Narrative Review on the Second Brain Aging. Front. Med..

[37] DiCarlo G., Wilcock D., Henderson D., Gordon M., Morgan D. (2001). Intrahippocampal LPS injections reduce Aβ load in APP+PS1 transgenic mice. Neurobiol. Aging.

[38] Herber D.L., Mercer M., Roth L.M., Symmonds K., Maloney J., Wilson N., Freeman M.J., Morgan D., Gordon M.N. (2007). Microglial Activation is Required for Aβ Clearance After Intracranial Injection of Lipopolysaccharide in APP Transgenic Mice. J. Neuroimmune Pharmacol..

[39] Alkasir R., Li J., Li X., Jin M., Zhu B. (2016). Human gut microbiota: The links with dementia development. Protein Cell.

[40] Main B.S., Minter M.R. (2017). Microbial Immuno-Communication in Neurodegenerative Diseases. Front. Neurosci..

[41] Zhang H., Chen Y., Wang Z., Xie G., Liu M., Yuan B., Cai H., Wang W., Cheng P. (2022). Implications of gut microbiota in neuro-degenerative diseases. Front. Immunol..

[42] Zhang Y., Yu W., Zhang L., Wang M., Chang W. (2022). The Interaction of Polyphenols and the Gut Microbiota in Neurodegenerative Diseases. Nutrients.

[43] Sun Y., Ho C.-T., Zhang X. (2023). Neuroprotection of Food Bioactives in Neurodegenerative Diseases: Role of the Gut Microbiota and Innate Immune Receptors. J. Agric. Food Chem..

[44] Rahman Z., Dandekar M.P. (2022). Implication of Paraprobiotics in Age-Associated Gut Dysbiosis and Neurodegenerative Diseases. NeuroMolecular Med..

[45] Borsom E.M., Conn K., Keefe C.R., Herman C., Orsini G.M., Hirsch A.H., Avila M.P., Testo G., Jaramillo S.A., Cope E.K. (2023). Predicting neu-rodegenerative disease using Prepathology gut microbiota composition: A longitudinal study in mice modeling Alzheimer’s disease pathologies. Microbiol. Spectr..

[46] Beker M.C., Caglayan B., Yalcin E., Caglayan A.B., Turkseven S., Gurel B., Kelestemur T., Sertel E., Sahin Z., Kutlu S. (2017). Time-of-Day Dependent Neuronal Injury After Ischemic Stroke: Implication of Circadian Clock Transcriptional Factor Bmal1 and Survival Kinase AKT. Mol. Neurobiol..

[47] Demircan T., Keskin I., Dumlu S.N., Aytürk N., Avşaroğlu M.E., Akgün E., Öztürk G., Baykal A.T. (2017). Detailed tail proteomic analysis of axolotl (*Ambystoma mexicanum*) using an mRNA-seq reference database. Proteomics.

[48] Harach T., Marungruang N., Duthilleul N., Cheatham V., Mc Coy K.D., Frisoni G., Neher J., Fåk F., Jucker M., Lasser T. (2017). Reduction of Abeta amyloid pathology in APPPS1 transgenic mice in the absence of gut microbiota. Sci. Rep..

[49] Emery D.C., Shoemark D.K., Batstone T.E., Waterfall C.M., Coghill J.A., Cerajewska T.L., Davies M., West N.X., Allen-Birt S.J. (2017). 16S rRNA Next Generation Sequencing Analysis Shows Bacteria in Alzheimer’s Post-Mortem Brain. Front. Aging Neurosci..

[50] Bradley W.G., Mash D.C. (2009). Beyond Guam: The cyanobacteria/BMAA hypothesis of the cause of ALS and other neuro-degenerative diseases. Amyotroph. Lateral Scler..

[51] Hu X., Wang T., Jin F. (2016). Alzheimer’s disease and gut microbiota. Sci. China Life Sci..

[52] Friedland R.P. (2015). Mechanisms of Molecular Mimicry Involving the Microbiota in Neurodegeneration. J. Alzheimer’s Dis..

[53] Schwartz K., Boles B.R. (2013). Microbial amyloids—Functions and interactions within the host. Curr. Opin. Microbiol..

[54] Branton W.G., Ellestad K.K., Maingat F., Wheatley B.M., Rud E., Warren R.L., Holt R.A., Surette M.G., Power C. (2013). Brain Microbial Populations in HIV/AIDS: α-Proteobacteria Predominate Independent of Host Immune Status. PLoS ONE.

[55] Païssé S., Valle C., Servant F., Courtney M., Burcelin R., Amar J., Lelouvier B. (2016). Comprehensive description of blood microbiome from healthy donors assessed by 16S targeted metagenomic sequencing. Transfusion.

[56] Jeong J.J., Kim K.A., Jang S.E., Woo J.Y., Han M.J., Kim D.H. (2015). Correction: Orally administrated Lactobacillus pentosus var. plantarum C29 ameliorates age-dependent colitis by inhibiting the nuclear factor-kappa B signaling pathway via the regulation of lipopolysaccharide production by gut microbiota. PLoS ONE.

[57] Hufnagel D.A., Tükel Ç., Chapman M.R. (2013). Disease to Dirt: The Biology of Microbial Amyloids. PLoS Pathog..

[58] Fransen F., Van Beek A.A., Borghuis T., El Aidy S., Hugenholtz F., van der Gaast–de Jongh C., Savelkoul H.F.J., De Jonge M.I., Boekschoten M.V., Smidt H. (2017). Aged Gut Microbiota Contributes to Systemical Inflammaging after Transfer to Germ-Free Mice. Front. Immunol..

[59] Bäuerl C., Collado M.C., Diaz Cuevas A., Viña J., Pérez Martínez G. (2018). Shifts in gut microbiota composition in an APP/PSS1 transgenic mouse model of Alzheimer’s disease during lifespan. Lett. Appl. Microbiol..

[60] Nagpal R., Neth B.J., Wang S., Craft S., Yadav H. (2019). Modified Mediterranean-ketogenic diet modulates gut microbiome and short-chain fatty acids in association with Alzheimer’s disease markers in subjects with mild cognitive impairment. Ebiomedicine.

[61] Miklossy J. (2015). Historic evidence to support a causal relationship between spirochetal infections and Alzheimer’s disease. Front. Aging Neurosci..

[62] Miklossy J. (2011). Alzheimer’s disease—A neurospirochetosis. Analysis of the evidence following Koch’s and Hill’s criteria. J. Neuroinflammation.

[63] Miklossy J. (2017). Alzheimer’s Disease, Spirochetes—A Causal Relationship. Innov. Aging.

[64] Lee H.-J., Hwang Y.-H., Kim D.-H. (2018). *Lactobacillus plantarum* C29-Fermented Soybean (DW2009) Alleviates Memory Impairment in 5XFAD Transgenic Mice by Regulating Microglia Activation and Gut Microbiota Composition. Mol. Nutr. Food Res..

[65] Broxmeyer L. (2017). Dr. Oskar Fischer’s Curious Little Alzheimer’s Germ. Curr. Opin. Neurol. Sci..

[66] La Rosa F., Clerici M., Ratto D., Occhinegro A., Licito A., Romeo M., Di Iorio C., Rossi P. (2018). The Gut-Brain Axis in Alzheimer’s Disease and Omega-3. A Critical Overview of Clinical Trials. Nutrients.

[67] Askarova S., Umbayev B., Masoud A.R., Kaiyrlykyzy A., Safarova Y., Tsoy A., Kushugulova A., Olzhayev F. (2020). The Links Between the Gut Microbiome, Aging, Modern Lifestyle and Alzheimer’s Disease. Front. Cell. Infect. Microbiol..

[68] Harach T., Marungruang N., Dutilleul N., Cheatham V., Mc Coy K.D., Neher J.J., Jucker M., Fåk F., Lasser, Bolmont T. (2015). Reduction of Alzheimer’s disease beta-amyloid pathology in the absence of gut microbiota. arXiv.

[69] Rinninella E., Raoul P., Cintoni M., Franceschi F., Miggiano G.A.D., Gasbarrini A., Mele M.C. (2019). What Is the Healthy Gut Microbiota Composition? A Changing Ecosystem across Age, Environment, Diet, and Diseases. Microorganisms.

[70] Arumugam M., Raes J., Pelletier E., Le Paslier D., Yamada T., Mende D.R., Fernandes G.R., Tap J., Bruls T., Batto J.M. (2011). Enterotypes of the human gut microbiome. Nature.

[71] Wang J., Lang T., Shen J., Dai J., Tian L., Wang X. (2019). Core Gut Bacteria Analysis of Healthy Mice. Front. Microbiol..

[72] Vital M., Howe A.C., Tiedje J.M. (2014). Revealing the Bacterial Butyrate Synthesis Pathways by Analyzing (Meta)genomic Data. mBio.

[73] Lukiw W.J. (2016). Bacteroides fragilis lipopolysaccharide and inflammatory signaling in Alzheimer’s Disease. Front. Microbiol..

[74] Zhao Y., Jaber V., Lukiw W.J. (2017). Secretory Products of the Human GI Tract Microbiome and Their Potential Impact on Alzheimer’s Disease (AD): Detection of Lipopolysaccharide (LPS) in AD Hippocampus. Front. Cell. Infect. Microbiol..

[75] Sergeant N., Wattez A., Galvn-Valencia M., Ghestem A., David J.-P., Lemoine J., Sautire P.-E., Dachary J., Mazat J.-P., Michalski J.-C. (2003). Association of atp synthase α-chain with neurofibrillary degeneration in alzheimer’s disease. Neuroscience.

[76] Terni B., Boada J., Portero-Otin M., Pamplona R., Ferrer I. (2010). Mitochondrial ATP-Synthase in the Entorhinal Cortex Is a Target of Oxidative Stress at Stages I/II of Alzheimer’s Disease Pathology. Brain Pathol..

[77] Beck S.J., Guo L., Phensy A., Tian J., Wang L., Tandon N., Gauba E., Lu L., Pascual J.M., Kroener S. (2016). Deregulation of mitochondrial F1FO-ATP synthase via OSCP in Alzheimer’s disease. Nat. Commun..

[78] Mi Y., Qi G., Brinton R.D., Yin F. (2021). Mitochondria-Targeted Therapeutics for Alzheimer’s Disease: The Good, the Bad, the Potential. Antioxid. Redox Signal..

[79] Mangione M.R., Vilasi S., Marino C., Librizzi F., Canale C., Spigolon D., Bucchieri F., Fucarino A., Passantino R., Cappello F. (2016). Hsp60, amateur chaperone in amyloid-beta fibrillogenesis. Biochim. Biophys. Acta.

[80] Singh N.K., Rao P., Asea A. (2008). Heat Shock Proteins and the Brain: Implications for Neurodegenerative Diseases and Neuroprotection. Heat Shock Proteins and the Brain: Implications for Neurodegenerative Diseases and Neuroprotection.

[81] Veereshwarayya V., Kumar P., Rosen K.M., Mestril R., Querfurth H.W. (2006). Differential Effects of Mitochondrial Heat Shock Protein 60 and Related Molecular Chaperones to Prevent Intracellular β-Amyloid-induced Inhibition of Complex IV and Limit Apoptosis. J. Biol. Chem..

[82] Walls K.C., Coskun P., Gallegos-Perez J.-L., Zadourian N., Freude K., Rasool S., Blurton-Jones M., Green K.N., LaFerla F.M. (2012). Swedish Alzheimer Mutation Induces Mitochondrial Dysfunction Mediated by HSP60 Mislocalization of Amyloid Precursor Protein (APP) and Beta-Amyloid. J. Biol. Chem..

[83] Mamelak M. (2007). Alzheimer’ s disease, oxidative stress and gammahydroxybutyrate. Neurobiol. Aging.

[84] Wang H., Wang Y., Hong X., Li S., Wang Y. (2016). Quantitative Proteomics Reveals the Mechanism of Oxygen Treatment on Lenses of Alzheimer’s Disease Model Mice. J. Alzheimer’s Dis..

[85] Lee T.-R., Lee H.-Y., Huang S.-H., Chan H.-T., Lyu P.-C., Chan H.-L. (2013). Comparative proteomics analysis of normal and memory-deficient Drosophila melanogaster heads. Zoöl. Stud..

[86] Lukiw W.J., Cho H.J., Kaufmann J.C., McLachlan D.R.C. (1990). The molecular mechanisms of scrapie encephalopathy and relevance to human neurodegenerative disease. Can. J. Veter. Res. Rev. Can. Rech. Veter..

[87] Lukiw W., Kruck T., McLachlan D. (1989). Linker histone-DNA complexes: Enhanced stability in the presence of aluminum lactate and implications for Alzheimer’s disease. FEBS Lett..

[88] Masters C.L., Bateman R., Blennow K., Rowe C.C., Sperling R.A., Cummings J.L. (2015). Alzheimer’s disease. Nat. Rev. Dis. Primers.

[89] Kumar D.K.V., Choi S.H., Washicosky K.J., Eimer W.A., Tucker S., Ghofrani J., Lefkowitz A., McColl G., Goldstein L.E., Tanzi R.E. (2016). Amyloid-β peptide protects against microbial infection in mouse and worm models of Alzheimer’s disease. Sci. Transl. Med..

[90] Pistollato F., Sumalla Cano S., Elio I., Masias Vergara M., Giampieri F., Battino M. (2016). Role of gut microbiota and nutrients in amyloid formation and pathogenesis of Alzheimer disease. Nutr. Rev..

[91] Gorevic P. (2014). Genetic Factors in the Amyloid Diseases.

[92] Deane R., Du Yan S., Submamaryan R.K., LaRue B., Jovanovic S., Hogg E., Welch D., Manness L., Lin C., Yu J. (2003). RAGE mediates amyloid-β peptide transport across the blood-brain barrier and accumulation in brain. Nat. Med..

[93] Paudel Y.N., Angelopoulou E., Piperi C., Othman I., Aamir K., Shaikh M.F. (2020). Impact of HMGB1, RAGE, and TLR4 in Alzheimer’s Disease (AD): From Risk Factors to Therapeutic Targeting. Cells.

[94] Shi D.Y., Bierhaus A., Nawroth P.P., Stern D.M. (2009). RAGE and Alzheimer’s disease: A progression factor for amyloid-β- induced cellular perturbation?. J. Alzheimer’s Dis..

[95] Allen H.B. (2016). Alzheimer’s Disease: Assessing the Role of Spirochetes, Biofilms, the Immune System, and Amyloid-β with Regard to Potential Treatment and Prevention. J. Alzheimer’s Dis..

[96] Lim J.-E., Kou J., Song M., Pattanayak A., Jin J., Lalonde R., Fukuchi K.-I. (2011). MyD88 Deficiency Ameliorates β-Amyloidosis in an Animal Model of Alzheimer’s Disease. Am. J. Pathol..

[97] Chen S.G., Stribinskis V., Rane M.J., Demuth D.R., Gozal E., Roberts A.M., Jagadapillai R., Liu R., Choe K., Shivakumar B. (2016). Exposure to the Functional Bacterial Amyloid Protein Curli Enhances Alpha-Synuclein Aggregation in Aged Fischer 344 Rats and Caenorhabditis elegans. Sci. Rep..

[98] Frost B., Diamond M.I. (2010). Prion-like mechanisms in neurodegenerative diseases. Nat. Rev. Neurosci..

[99] Apajalahti J.H.A., Särkilahti L.K., Mäki B.R., Heikkinen J.P., Nurminen P.H., Holben W.E. (1998). Effective Recovery of Bacterial DNA and Percent-Guanine-Plus-Cytosine-Based Analysis of Community Structure in the Gastrointestinal Tract of Broiler Chickens. Appl. Environ. Microbiol..

[100] Tanca A., Palomba A., Pisanu S., Deligios M., Fraumene C., Manghina V., Pagnozzi D., Addis M.F., Uzzau S. (2014). A straightforward and efficient analytical pipeline for metaproteome characterization. Microbiome.

[101] Ngo S., Guo Z. (2011). Key residues for the oligomerization of Aβ42 protein in Alzheimer’s disease. Biochem. Biophys. Res. Commun..

[102] Wiśniewski J.R., Zougman A., Nagaraj N., Mann M. (2009). Universal sample preparation method for proteome analysis. Nat. Methods.

[103] Singh R.G., Tanca A., Palomba A., Van Der Jeugt F., Verschaffelt P., Uzzau S., Martens L., Dawyndt P., Mesuere B. (2018). Unipept 4.0: Functional Analysis of Metaproteome Data. J. Proteome Res..

