# Supplementary material for: Bridging the Gap between Gut Microbiota and Alzheimer’s Disease: A Metaproteomic Approach for Biomarker Discovery in Transgenic Mice
Source: Int J Mol Sci. 2023 Aug 15;24(16):12819. doi: 10.3390/ijms241612819 (PMC10454110; doi:10.3390/ijms241612819)
Supplement: Supplementary file 1 [file ijms-24-12819-s001.zip › ijms-2506029-supplementary.pdf]

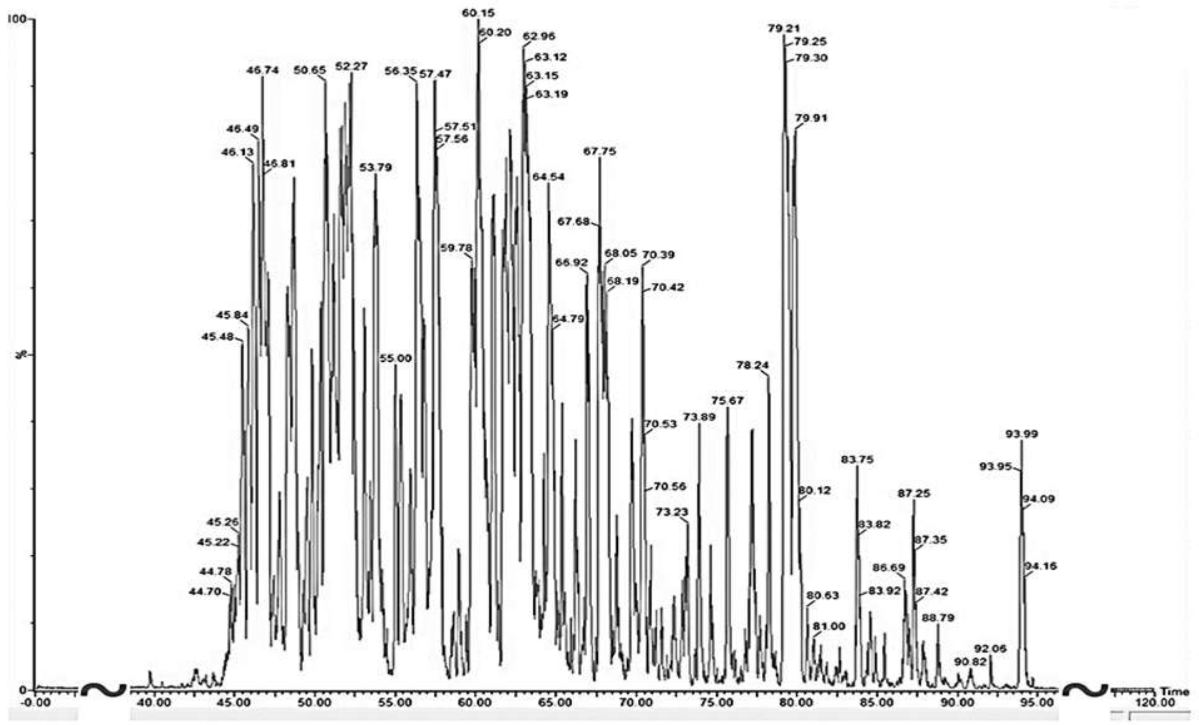

Supplementary Figure S1. A representative LC-MS/MS chromatogram of the metaproteome in the murine model used in the study.

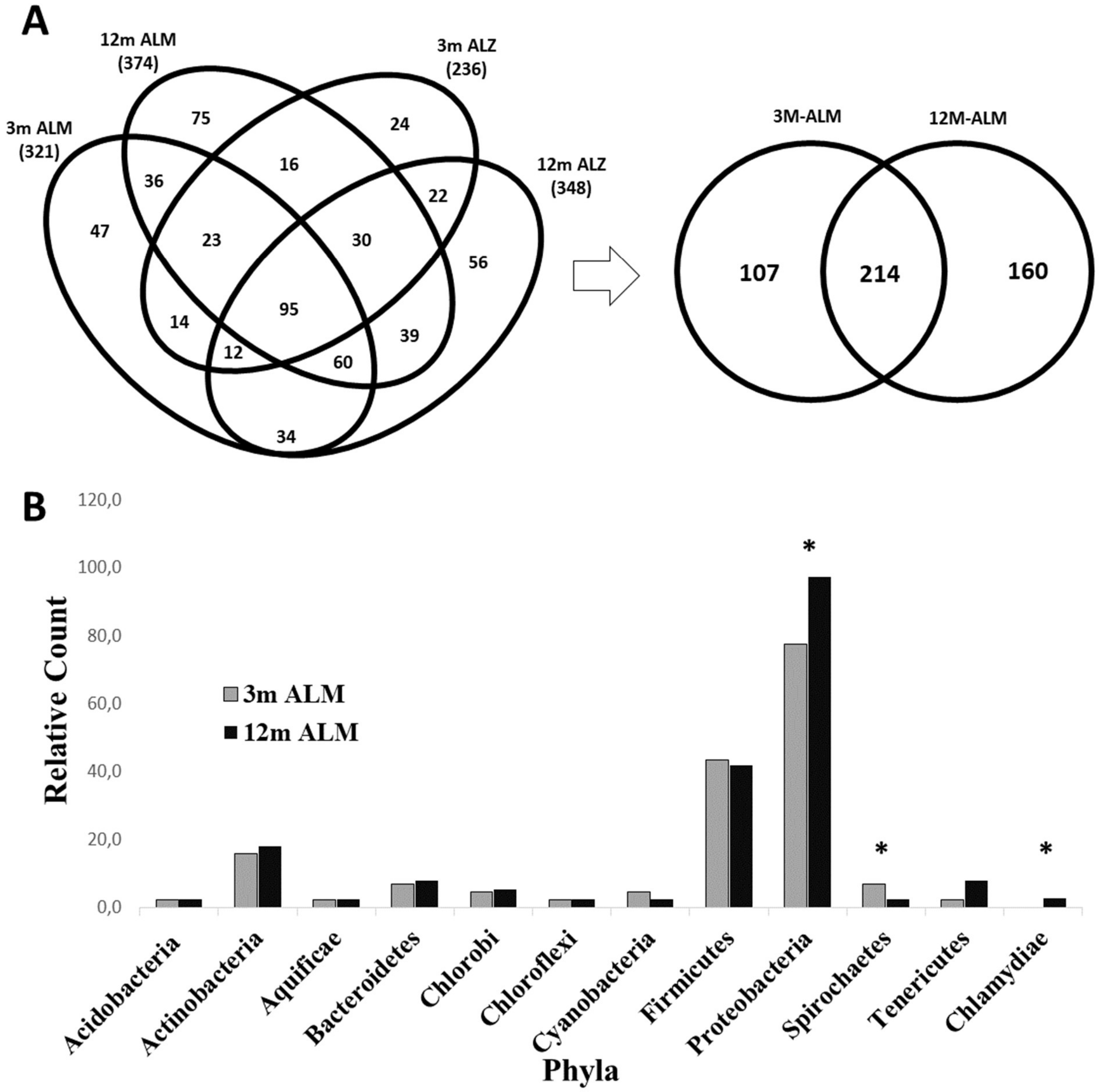

Supplementary Figure S2. (A) Venn diagram of normal aging process based on the number of organisms. (C) The bar graph displays changes in the abundance of different phyla. Statistical analysis using the *MedCalc-Test for one proportion* indicates that phyla have significantly increased or decreased in proportion. Proteobacteria: p = 0.0012, Spirochaetes: p = 0.0241, Chlamydiae: p = 0.0207. * P <0.05.

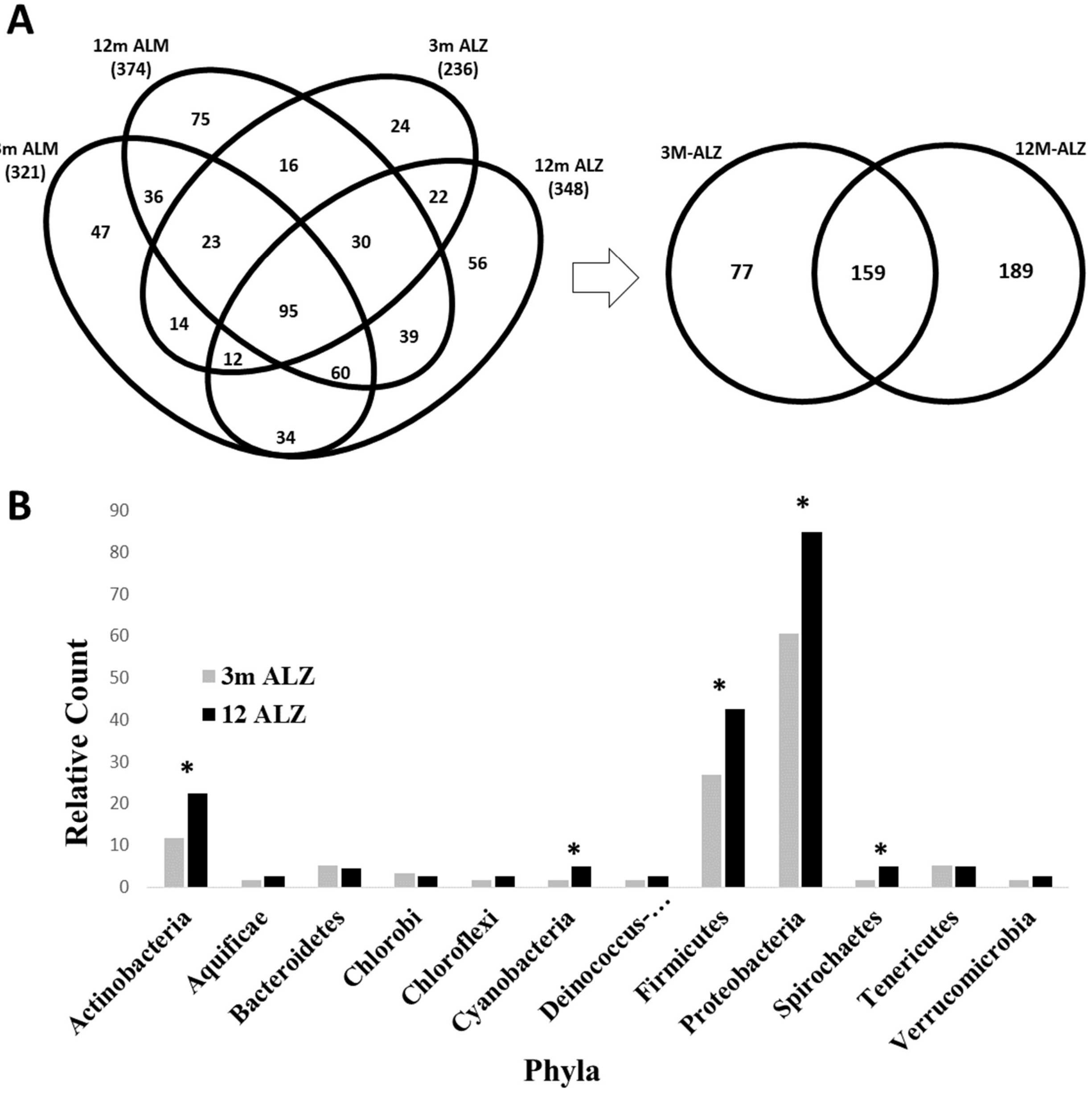

Supplementary Figure S3. The aging microbial changes in Tg cohorts illustrated by the Venn diagram to assess the number of organisms involved, presenting in (A). The bar graph used to analyze changes in the abundance of different phyla during the aging duration (B). Statistical analysis using the *MedCalc-Test for one proportion* revealed that certain phyla had significantly increased or decreased in proportion. Actinobacteria: p=0,0114; Cyanobacteria: p=0,0092; Firmicutes: p=0,0056; Proteobacteria: p = 0.0008; Spirochaetes: p = 0.0092. The significance level for these changes is denoted by * P <0.05.

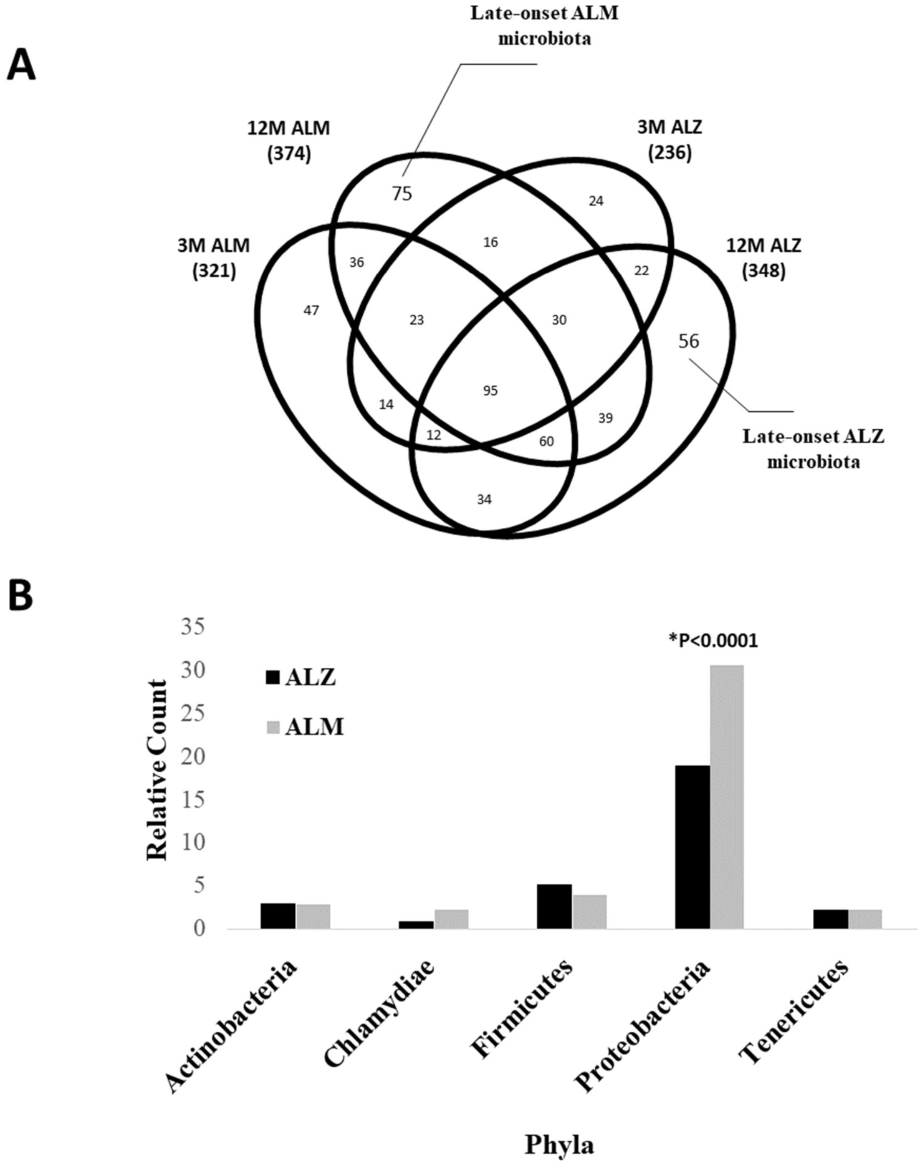

Supplementary Figure S4. (A) Representation of the Venn diagram depicting the number of unique organisms in 12M ALM and 12M ALZ microbiota. (B) Displaying the bar graph version of the phylum diagram, which indicated the percentage of each phylum, and a statistical analysis using the *MedCalc-Test for one proportion*. The results reveal a significant change in the abundance of *Proteobacteria* ($p<0.0001$).
